# Imaging findings of Budd-Chiari syndrome caused by intravenous leiomyomatosis: a case report

**DOI:** 10.3389/fmed.2024.1422652

**Published:** 2024-08-13

**Authors:** Yahong Wang, Yifan Dong, Xueyu Tian, Yu Chen, Xiao Yang, Jianchu Li

**Affiliations:** ^1^Department of Medical Ultrasound, Peking Union Medical College Hospital, Chinese Academy of Medical Sciences and Peking Union Medical College, Beijing, China; ^2^Department of Ultrasound, Tianjin Wuqing District Hospital of Traditional Chinese Medicine, Tianjin, China; ^3^Department of Radiology, Peking Union Medical College Hospital, Chinese Academy of Medical Sciences and Peking Union Medical College, Beijing, China

**Keywords:** case report, intravenous leiomyomatosis, Budd-Chiari syndrome, computed tomography, ultrasonography, imaging

## Abstract

Intravenous leiomyomatosis (IVL) is a rare gynecological-related tumor. It can invade and extend in the blood vessel and eventually involve the cardiac cavity or even the pulmonary artery. IVL generally does not adhere to the vein wall and infrequently leads to the manifestation of Budd-Chiari syndrome (BCS). In this case report, the presence of a sizable tumor obstructed the second hepatic portal, impeding the return flow of the hepatic veins, thereby precipitating the development of BCS. The presence of collateral veins and dilation of the accessory hepatic vein were identified through computed tomography venography and ultrasonography, thus supporting the diagnosis of BCS. The patient underwent a comprehensive surgical resection and was found to have a favorable prognosis.

## Introduction

Intravenous leiomyomatosis (IVL) is known as a rare intravascular disease that may be localized within the pelvic region or extend into the inferior vena cava, cardiac cavity or even pulmonary artery, with a high risk of sudden death and pulmonary embolism (1, 2). It may present asymptomatically or with symptoms such as abnormal uterine bleeding, chest distress, dyspnea, and lower limb edema (3). However, there have been few reports of Budd-Chiari syndrome (BCS) caused by IVL. In this report, we present a rare case of a giant IVL, obstructing the second hepatic portal, impeding the return flow of the hepatic veins, thereby precipitating the development of BCS. It is worth noting that the concurrent use of computed tomography and ultrasonography offers significant benefits in the diagnosis and assessment of this condition.

## Case presentation

A 49-year-old female was admitted to the local hospital 1 month ago due to recurrent vaginal bleeding. Thirteen years prior, the patient had undergone a myomectomy procedure. Subsequent gynecological ultrasonography identified multiple uterine fibroids, one of which was located beneath the mucosa and measured 3.4 cm × 3.7 cm × 2.0 cm. As a result, the patient was scheduled for surgical intervention. However, preoperative echocardiography revealed the presence of space-occupying lesions in the right atrium and inferior vena cava, leading to a referral to our hospital. During the physical examination, it was observed that the heart sound exhibited low intensity and weakness. Additionally, no pathological murmurs were detected during auscultation of each valve area, and no tumor-related fluttering sounds were present. Below the umbilicus and above the pubic symphysis, palpation revealed firm and less mobile mass located in the area between the outer margin of the rectus abdominis on both sides. This mass exhibited a regular shape and displayed distinct boundary with the surrounding tissues.

**FIGURE 1:**
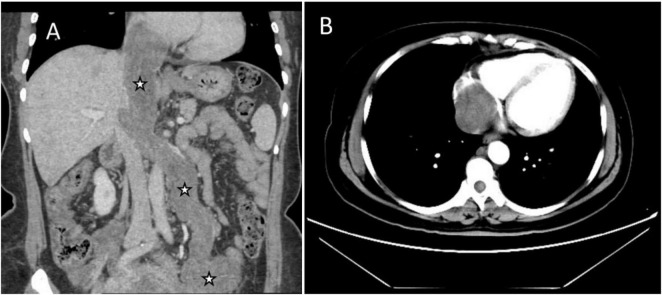
Images of the mass in inferior vena cava (IVC) and right atrium on enhanced CT. **(A)** Coronal image showed multiple soft tissue masses surrounding the uterus, extending from the left ovarian vein, left renal vein, and IVC to the right atrium (asterisks). **(B)** Transverse section showed that the lesion occupies almost the entire right atrium.

**FIGURE 2:**
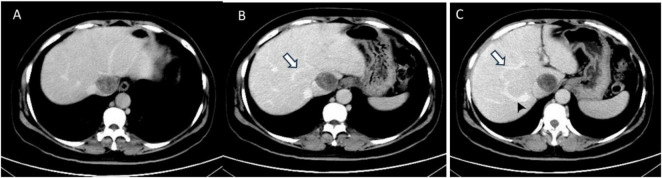
The transverse sections of enhanced abdominal CT images. **(A)** The second hepatic hilum was obstructed by the mass. **(B,C)** Arrows demonstrated the presence of communicating branches between the hepatic veins. **(C)** The arrow head pointed the dilated accessory hepatic vein.

Abdominal enhanced computed tomography (CT) and inferior vena cava CT venography (CTV) demonstrated the presence of multiple soft tissue masses surrounding the uterus, extending from the left ovarian vein, left renal vein, and inferior vena cava to the right atrium (Figure 1). These masses exhibited slight enhancement. The second hepatic hilum was obstructed by the mass, resulting in a slender middle hepatic vein and a dilated accessory hepatic vein in the posterior right lobe. Additionally, the formation of communicating branches between the hepatic veins was observed (Figure 2).

**FIGURE 3:**
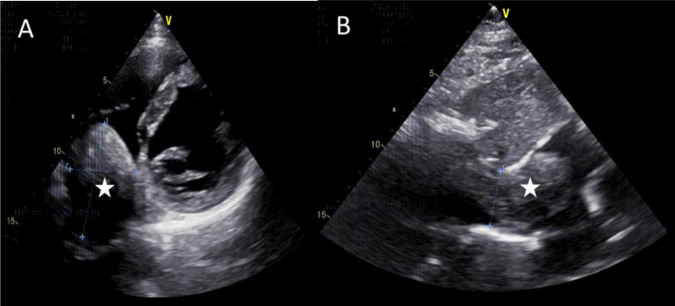
Images of transthoracic echocardiogram. **(A,B)** The asterisks indicated the hypoechoic mass located within the right atrium and inferior vena cava, measuring 6.3 cm × 4.8 cm in dimensions. **(B)** The inferior vena cava exhibited dilation with a proximal diameter of 3.1 cm.

**FIGURE 4:**
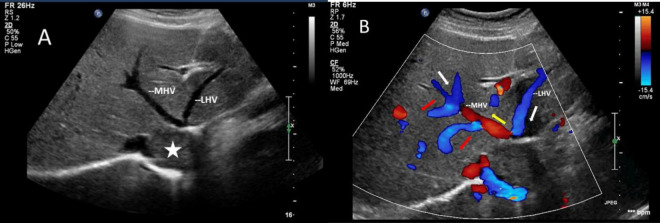
Images of liver greyscale and color Doppler sonography. **(A)** The lesion (asterisk) obstructed the blood flow from the left and middle hepatic veins to the inferior vena cava, resulting in a reversal of blood flow from the left hepatic vein into the middle hepatic vein. **(B)** Arrows show the direction of the blood flow: the white arrows illustrate the normal direction of blood flow, while the yellow arrows indicate the retrograde flow of the proximal segment of MHV, which drains LHV and redirects it through collateral vessels (red arrows) to the veins in the right lobe of the liver. LHV, left hepatic vein; MHV, middle hepatic vein.

**FIGURE 5:**
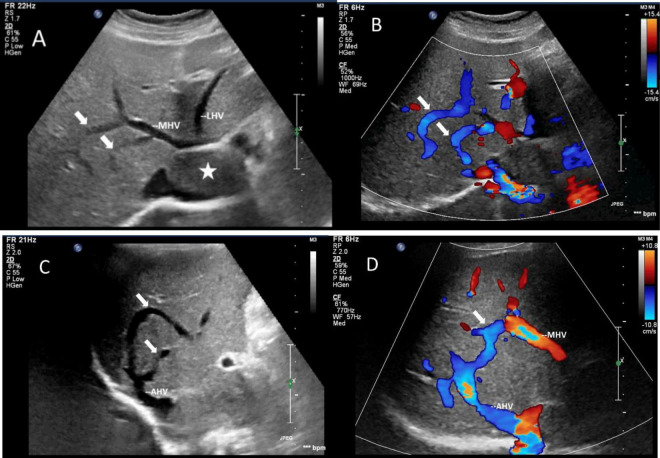
Images of collateral circulation between hepatic veins on greyscale and color Doppler sonography. White arrows indicated the collateral veins. **(A,C)** Gray-scale images displayed the collateral veins connecting MHV and the dilated accessory hepatic vein in the posterior right lobe from various perspectives. **(B,D)** Color Doppler blood flow images illustrated the blood flow within the collateral veins from different angles. The blood flow direction was observed to move from MHV toward the dilated accessory hepatic vein, ultimately draining into the inferior vena cava. The asterisk denoted the presence of a tumor. LHV, left hepatic vein; MHV, middle hepatic vein; AHV, accessory hepatic vein.

The mass can also be visualized within the right atrium and inferior vena cava using gray scale ultrasound imaging, appearing as a hypoechoic lesion of considerable length (Figure 3). Additionally, color Doppler ultrasonography can be utilized to assess hepatic vein obstruction and the direction of hepatic blood reflux. We can see that there is obstruction in the blood flow of the left and middle hepatic veins, resulting in a reversal of blood flow from the left hepatic vein into the middle hepatic vein (Figure 4). This obstruction has led to the formation of multiple collateral circulations between the middle hepatic vein and either the right hepatic vein or the dilated accessory hepatic vein in the posterior right lobe (Figure 5). These collateral circulations serve to drain the blood flow from the distal segment of the middle hepatic vein and the left hepatic vein into the inferior vena cava.

The patient underwent a hysterectomy, bilateral adnexectomy, combined thoracoabdominal exploration, and resection of intravenous masses. During the surgical procedure, it was determined that the leiomyoma originated from the left side, extended along the left reproductive vein, and subsequently merged into the left renal vein, inferior vena cava, and right atrium. The postoperative pathological diagnosis revealed multiple uterine leiomyoma with intravenous leiomyomatosis. The patient experienced a successful recovery with no recurrence observed for a period of 7 years.

## Discussion

Intravenous leiomyomatosis (IVL) is a rare intravascular disease characterized by histological benignity but exhibiting a biological behavior akin to malignancy (4, 5). It predominantly affects women in the reproductive and perimenopausal age groups, most of whom have a history of uterine fibroids, and commonly diagnosed in the fourth decade of life (2). IVL can arise from extravascular leiomyomas, such as uterine fibroids, and infiltrate vascular structures by traversing through the muscular walls into the parauterine vein. Alternatively, IVL may originate from the smooth muscle of vascular walls and extend into the lumen (4, 5). While IVL may be localized within the uterus, it can also disseminate into pelvic veins, including the iliac or ovarian veins, ultimately reaching the inferior vena cava, right atrium, or potentially traversing the tricuspid valve into the right ventricle (2, 6).

IVL generally does not adhere to the vein wall and infrequently leads to the manifestation of Budd-Chiari syndrome. Only three case reports were identified when utilizing the search terms Budd-Chiari syndrome and intravenous leiomyomatosis on PubMed (7–9). The tumors in the three patients were found to be extensive, reaching the inferior vena cava and right atrium. Two patients reported by Barksdale et al. and Kuenen et al. exhibited acute BCS, marked by acute thrombosis of three hepatic veins without collateral circulation, with no ultrasound assessment conducted (7, 8). The third case reported by Park et al. presented with chronic BCS and collateral circulation resulting from focal obstruction at the hepatic venous entrance, mirroring our own case, yet lacking hemodynamic evaluation via color Doppler ultrasound (9).

In this case, the presence of a sizable tumor obstructed the second hepatic portal, impeding the return flow of the left and right hepatic veins, thereby precipitating the development of BCS. This variant of BCS often leads to the formation of collateral circulation routes linking the hepatic vein (HV) and the inferior vena cava, including HV-accessory HV collaterals, HV-HV collaterals, HV-accessory HV plus HV collaterals, among others (10). The most prevalent form is HV-accessory HV collaterals, with the accessory HVs encompassing the caudate lobe veins and inferior right HVs. This results in the dilation of the caudate lobe veins and inferior right HVs, particularly the inferior right hepatic vein, along with hypertrophy of the caudate lobe. The hepatic vein reflux disorder can subsequently lead to portal hypertension, manifesting in hepatosplenomegaly, ascites, varices, and other clinical manifestations (11).

CTV is capable of accurately assessing the growth trajectory, size, and extent of involvement of IVL tumors within blood vessels. IVL typically presents as soft tissue density within the vein on CTV, exhibiting heterogeneous contrast enhancement, frequently in conjunction with a background of multiple uterine fibroids or prior resection of multiple uterine fibroids. The luffa sponge sign or sieve sign is a typical manifestation on enhanced CT (12). The tumor demonstrates a tendency to grow cephalad along the iliac vein or ovarian vein, eventually infiltrating the inferior vena cava and potentially extending into the right atrium, leading to obstruction of the right heart. Ultrasound imaging enables real-time observation of tumors and assessment of hemodynamics, including tumor activity in the right atrium and inferior vena cava, as well as evaluation of right heart function, iliac vein obstruction, and hepatic vein obstruction. The combined use of CTV and ultrasound allows for a comprehensive evaluation of the tumor and accurate diagnosis of secondary Budd-Chiari syndrome. This approach also provides clear indications of hemodynamic changes in the liver, facilitating precise disease evaluation.

IVL should be differentiated primarily from deep vein thrombosis, inferior vena cava leiomyosarcoma, and tumor thrombus originating from retroperitoneal malignant tumors. Large-scale deep venous thrombosis typically presents with predisposing factors such as thrombophilia, with the thrombus and venous wall position being relatively stable. In cases of chronic thrombosis with recanalization, the thrombus is often adherent to the venous wall and lacks nourishing blood vessels, resulting in a lack of enhancement on CT imaging. Leiomyosarcoma of the inferior vena cava typically presents as segmental involvement, with infrequent extension to the right atrium (13). These lesions originate from the vascular wall, often exhibiting an indistinct boundary with the venous wall, and may invade into surrounding tissues such as the liver and peripheral structures. Tumor thrombi originating from retroperitoneal malignant tumors may arise from renal cell carcinoma or adrenal malignant tumors, with affected patients presenting either primary tumor lesions or relevant medical histories. The tumor thrombus associated with leiomyosarcoma or other malignant tumors in the inferior vena cava typically exhibits irregular shape, abundant and disorganized blood flow within the solid component, and uneven enhancement on enhanced CT imaging. Contrast-enhanced ultrasound (CEUS) can also reveal the microvascular perfusion within the lesion, with IVL demonstrating a distinct sieve hole sign or multi-track sign on CEUS, aiding in the differentiation from other pathologies (14).

In summary, intravenous leiomyomatosis is a tumor that exhibits unique characteristics in terms of its biological behavior and imaging features, and can lead to secondary complications such as Budd-Chiari syndrome. The utilization of multiple imaging modalities is beneficial for the accurate diagnosis and assessment of IVL.

## Data availability statement

The original contributions presented in this study are included in this article/supplementary material, further inquiries can be directed to the corresponding authors.

## Ethics statement

The studies involving humans were approved by the Institutional Review Board of Peking Union Medical College Hospital. The studies were conducted in accordance with the local legislation and institutional requirements. The participants provided their written informed consent to participate in this study. Written informed consent was obtained from the individual(s) for the publication of any potentially identifiable images or data included in this article.

## Author contributions

YHW: Conceptualization, Data curation, Formal analysis, Funding acquisition, Investigation, Methodology, Project administration, Resources, Software, Supervision, Validation, Visualization, Writing – original draft, Writing – review and editing. YFD: Writing – original draft. XYT: Writing – original draft. YC: Writing – review and editing. XY: Writing – review and editing. JCL: Writing – review and editing.

## References

[1] Hayashi T Yaegashi N Konishi I. Molecular pathological approach of uterine intravenous leiomyomatosis. *Ann Transl Med.* (2022) 10:724–8. 35957719 10.21037/atm-22-2804 PMC9358516

[2] Lim W Lamaro V Sivagnanam V. Manifestation and management of intravenous leiomyomatosis: A systematic review of the literature. *Surg Oncol.* (2022) 45:101879. 10.1016/j.suronc.2022.101879 36332557

[3] Shi P Xiao H Li H Tang W Ren A Ma L Management and prognosis comparison between incidental and nonincidental intravenous leiomyomatosis: A retrospective single-center real-life experience. *Ann Transl Med.* (2022) 10:503–14. 10.21037/atm-21-5376 35692495 PMC9179019

[4] Valdés Devesa V Conley C Stone W Collins J Magrina J. Update on intravenous leiomyomatosis: Report of five patients and literature review. *Eur J Obstet Gynecol Reprod Biol.* (2013) 171:209–13. 10.1016/j.ejogrb.2013.09.031 24207051

[5] Grella L Arnold T Kvilekval K Giron F. Intravenous leiomyomatosis. *J Vasc Surg.* (1994) 20:987–94. 7990195 10.1016/0741-5214(94)90237-2

[6] Ahmed M Zangos S Bechstein W Vogl T. Intravenous leiomyomatosis. *Eur Radiol.* (2004) 14:1316–7. 14710312 10.1007/s00330-003-2186-z

[7] Barksdale J Abolhoda A Saremi F. Intravenous leiomyomatosis presenting as acute Budd-Chiari syndrome. *J Vasc Surg.* (2011) 54:860–3. 10.1016/j.jvs.2011.03.261 21620629

[8] Kuenen B Slee P Seldenrijk C Wagenaar S. Intravenous leiomyomatosis complicated by Budd-Chiari syndrome. *Postgrad Med J.* (1996) 72:686–8. 8944214 10.1136/pgmj.72.853.686 PMC2398644

[9] Park S Yeo I Kim Y Kim J. Obstruction of the hepatic venous flow caused by intravenous leiomyomatosis. *Medicina (Kaunas).* (2020) 56:696. 10.3390/medicina56120696 33327445 PMC7764919

[10] Cai S Gai Y Liu Q. Computed tomography angiography manifestations of collateral circulations in Budd-Chiari syndrome. *Exp Ther Med.* (2015) 9:399–404. 10.3892/etm.2014.2125 25574205 PMC4280983

[11] Valla D. Budd-Chiari syndrome/hepatic venous outflow tract obstruction. *Hepatol Int.* (2018) 12:168–80. 28685257 10.1007/s12072-017-9810-5

[12] Wang H Nie P Chen B Hou F Dong C He F Contrast-enhanced CT findings of intravenous leiomyomatosis. *Clin Radiol.* (2018) 73:503.e1–503.e6. 10.1016/j.crad.2017.12.016 29395222

[13] Mingoli A Feldhaus R Cavallaro A Stipa S. Leiomyosarcoma of the inferior vena cava: analysis and search of world literature on 141 patients and report of three new cases. *J Vasc Surg.* (1991) 14:688–99. 10.1067/mva.1991.30426 1942380

[14] Ge Z Wang Y Wang Y Fang S Wang H Li J. Diagnostic value of contrast-enhanced ultrasound in intravenous leiomyomatosis: A single-center experiences. *Front Oncol.* (2022) 12:963675. 10.3389/fonc.2022.963675 36033528 PMC9403056

